# Stigmata of Maize

**Published:** 1880-10

**Authors:** 


The Stigmata of Maize.—We copy from the London Practitioner's translation from the "Progres Medicale" the following on *corn-silk* as a diuretic. Some months ago we asked our readers to investigate this new remedy when the roasting-ear season came. It is now with us, and we hope some of our friends will report to us the result of their trials with it.

It is hardly a year since this remedy was first introduced into the ordinary routine of practice, and yet it may not be uninteresting to make an abstract of some of the papers which have been published in regard to it. Prof. Castan, at the Montpellier meeting, called attention to the stigmata of maize as a remedy which he had long known and which he had found to be of great use in gravel and nephritic colic. In the latter disease there ensued, after the administration of the drug, a marked decrease in the painful symptoms, and he, therefore, supposed that the stigmata acted less as a diuretic than a local anesthetic. Prof. Denuce, of Bordeux, obtained the most favorable results from it in vesical catarrh, in which it appears to pessess an elective action on the mucous membrane of the bladder. Dr. Pons, of Nerac, and Dr. Queirel, of Marseilles, had also frequently employed the stigmata of maize. M. Queirel observed that the pain was greatly alleviated in nephritic colic after the use of the remedy, but the urine was at the same time remarkably increased in quantity. At the Therapeutic Society M. Constantin Paul stated that he was not convinced of the diuretic properties of the stagmata, although one of his colleagues had obtained some very striking results, the quantity of urine being in one case of dropsy increased from five to fifteen hundred grains after the ingestion of three spoonfuls of the syrup. Dr. Landrieux has arrived at the following conclusions, based on a considerable number of observations:

1. The various preparations of the stigmata of maize are of use in modifying the secretions of the urinary tracts. They may also be considered to possess a distinctly diuretic action.

2. Diuresis is rapidly produced and the increase of urine is very marked after three or four days.

3. The diuretic effects are observed not only in diseases of the organs connected in the urinary section, but also in the affections of the vascular system (disease of the heart, blood vessels, etc.)

4. The pulse is regular, the arterial tension is increased, while the venous pressure is diminished.

5. The remedy produces no disturbance of the nervous or digestive systems. The tolerance of the drug is complete and absolute, while in chronic cases its administration may be continued for three to six months without inconvenience. The different results which the use of the stigmata of maize has given at the hands of different observers appears to be due in a large measure to the fact that the strength of the extract varies according to the nature of the soil, to the climate, to the time, to the mode of picking and to the manner of drying the stigmata. The formula for the preparation of the syrup is not yet fixed, since the quantity of the active principle varies in different samples of the stigmata. The Pharmaceutical Union adopts formulæ which contains in one case six and in another twelve grams of extract to the kilogram of syrup. The latter receipt is based on the assumption of a strength of 12 per cent. This quantity appears, however, to be too small, since the best samples of stigmata yield 25 to 30 per cent. of extract, or on an average 27.5 per cent. The kilogram of syrup will, therefore, contain 27.5 grams with this strength (27.5 pro mille). The daily dose of the syrup will be two to four spoonfuls, representing about one to two grams of the extract. In all cases the syrup should be employed in preference to an infusion of the stigmata of maize.—*Louisville Medical News*, July 3, 1880.

